# Fontaine progeroid syndrome with neonatal mitochondrial disease

**DOI:** 10.1038/s41439-025-00331-1

**Published:** 2025-11-21

**Authors:** Mitsuhiko Riko, Daiki Kawamoto, Kentaro Hirayama, Tomoya Tsuchihashi, Takayuki Suzuki, Takuya Sugimoto, Yasushi Okazaki, Kei Murayama, Daisuke Tokuhara

**Affiliations:** 1 Department of Pediatrics, Wakayama Medical University, Wakayama, Japan; 2 Diagnostics and Therapeutics of Intractable Diseases, Intractable Disease Research Center, Graduate School of Medicine, Juntendo University, Bunkyo, Tokyo, Japan

**Keywords:** Gene expression, Genetic variation

## Abstract

Fontaine progeroid syndrome (FPS) is a rare condition characterized by abnormalities in *SLC25A24*. Some instances of FPS have been reported to be fatal early in life. Here we present the first case of mitochondrial disease diagnosed with FPS in Japan. The diagnosis was based on the presence of the heterozygous known pathogenic variant of *SLC25A24*, NM_013386.5: c.649C>T and decreased activity of mitochondrial respiratory chain enzyme activity.

## First Japanese case of fontaine progeroid syndrome reported

A Japanese girl was born prematurely with no family history of genetic disorders. Doctors noticed her growth was slow during pregnancy, leading to an emergency delivery. After birth, she had severe health issues, including breathing problems and high levels of lactic acid in her blood. Despite intensive care and various treatments, her condition did not improve and she passed away at 15 days old. Doctors suspected a mitochondrial disorder and performed genetic testing. They found a mutation in the *SLC25A24* gene, which is linked to Fontaine progeroid syndrome, a rare genetic disorder causing early aging symptoms. This mutation was not inherited from her parents. The study highlights the importance of genetic testing in diagnosing rare conditions such as Fontaine progeroid syndrome. The findings suggest that certain mutations in the *SLC25A24*gene can lead to severe health issues from birth. This summary was initially drafted using artificial intelligence, then revised and fact-checked by the author.

Fontaine progeroid syndrome (FPS) is an autosomal dominant condition resulting from an abnormality in *SLC25A24*, with only 12 reported cases so far^1,2^. No cases have been reported in Japan. Various deformities have been documented, and while some patients survive until their teens, early death has also been reported. As *SLC25A24* is associated with the transport of electron-transfer system metabolites in the mitochondrial inner membrane, genetic abnormalities in this gene have been demonstrated to cause secondary oxidative phosphorylation defects^3^. Mitochondrial respiratory chain disorders (MRCD) can occur across all age groups, with about 16% of cases occurring in the neonatal period, half of which are reported to be fatal^3^.

Here, we report the first Japanese case of FPS with MRCD during the neonatal period. The patient was diagnosed with a pathogenic variant of *SLC25A24* that decreases mitochondrial respiratory chain complex enzyme activity.

A Japanese girl, born preterm as the second child to healthy parents with a healthy older son, had no family history of pathogenicity. Her parents are not in a consanguineous marriage. Her mother had a spontaneous pregnancy managed by a local obstetrician. Fetal growth restriction based on estimated fetal weight gradually worsened, from −1.0 s.d. at 19 weeks to −3.2 s.d. at 29 weeks of gestation. Consequently, she was referred to our tertiary hospital at 29 weeks and 5 days of gestation, leading to an emergency cesarean section owing to fetal insufficiency at 30 weeks and 0 days of gestation. The patient’s Apgar scores were 7 at 1 min and 8 at 5 min. Her birth weight was 767 g (−3.2 s.d.) and her birth height was 33.5 cm (−2.6 s.d.).

Prompt intensive care, including ventilatory management for an extremely low birth weight (ELBW) infant, was initiated in our neonatal intensive care unit. The patient presented severe pulmonary hypertension, hyperlactatemia and lactic acidosis in the early postnatal period. Despite initiating therapies such as nitric oxide inhalation, catecholamine and lipo-prostaglandin E1 (lipo-PGE1) for persistent pulmonary hypertension of the newborn (PPHN) and sodium hydrogen carbonate for acidosis, there was no improvement in PPHN or hyperlactatemia. The lactate/pyruvate ratio on day 1 was 23, raising suspicion of MRCD. Peritoneal dialysis was performed on day 1; however, the effect was poor. A vitamin cocktail therapy (coenzyme Q10 5 mg/kg/d, l-carnitine 100 mg/kg/d, vitamin E 10 mg/kg/d, thiamine 5 mg/kg/d, vitamin C 50 mg/kg/d and vitamin B complex) was subsequently introduced, but the hyperlactatemia and PPHN did not improve. The days passed without any improvement in her general condition, and by day 11, she developed sepsis and died on day 15.A pathological autopsy, conducted with parental consent, revealed decreased complex I and IV activity in muscle, especially complex I activity below 20% (Table 1). In the heart, complex I was slightly low but did not meet diagnostic criteria.

**Table 1:** Respiratory chain enzyme assay of the study patient.

	Co I	Co II	Co II + III	Co III	Co IV	CS
Muscle						
Percentage of normal	11.9	54.0	35.7	60.1	25.3	103.5
CS ratio (%)	11.5	53.1	34.1	59.5	23.1	
Co Ⅱ ratio (%)	21.1		62.4	109.9	41.0	
Heart						
Percentage of normal	20.0	36.2	70.0	45.9	41.0	52.4
CS ratio (%)	36.9	68.3	126.7	84.6	77.8	
Co Ⅱ ratio (%)						

*Co I* complex I, *Co II* complex II, *Co III* complex III, *Co IV* complex IV, *CS* citrate synthase.

Given the clinical and biochemical suspicion of mitochondrial disorder, we next performed targeted gene panel sequencing of genomic DNA extracted from peripheral blood. This in-house panel was designed for genetic testing of mitochondrial disease and developed with targets on the exons of 367 nuclear genes based on previous review articles^4,5^, as well as the entire 16.6-kb mitochondrial genome.

**Fig. 1:**
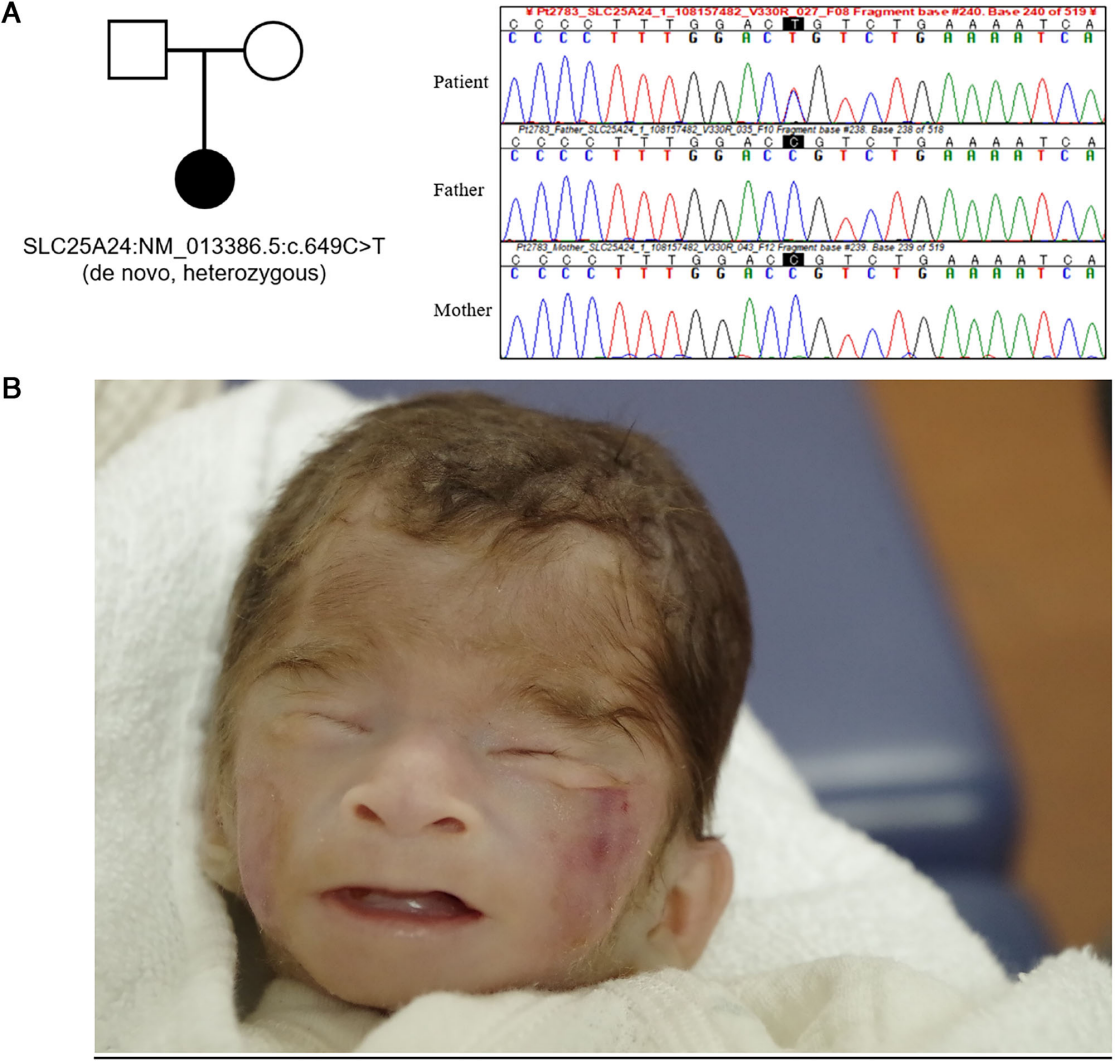
Sanger seqencing and clinical presentation of Fontaine progeroid syndrome. **A** A family tree and electropherogram of Sanger sequencing. **B** Facial characteristics of FPS. The facial features of the patient include hypertrichosis, depressed nasal bridge, low-set ears, thin upper lip.

The targeted resequencing described above identified a heterozygous variant, NM_013386.5:c.649C>T, p.(Arg217Cys), in exon 5 of *SLC25A24*, classified as pathogenic in FPS by ClinVar. As her parents did not carry this variant, the diagnosis was de novo FPS (Fig. 1A). According to the American College of Medical Genetics and Genomics guidelines^6^, this variant is regarded as pathogenic (1 strong (PS2), 3 moderate (PM1, PM2 and PM4)).

FPS is an autosomal dominant inherited condition caused by *SLC25A24*. A diverse range of symptoms has been documented with this condition, and the symptoms of the study patient case were consistent with several of these symptoms. The patient exhibited intrauterine growth restriction, translucent skin, prominent veins, hypertrichosis, decreased subcutaneous fat, depressed nasal bridge, low-set ears, thin upper lip (Fig. 1B) and congenital heart diseases, such as patent ductus arteriosus and pulmonary artery hypertension. These symptoms are consistent with those of previously reported cases^1,2^. No craniosynostosis was observed.

*SLC25A24* encodes a mitochondrial inner membrane ATP-Mg/Pi carrier known as short Ca^2+^-binding mitochondrial carrier 1. It comprises an N-terminal calcium-binding domain, six transmembrane helices and a short C-terminus^7^. The main function of ATP-Mg/Pi carriers involves the exchange of ATP-Mg or ADP for phosphate across the inner mitochondrial membrane. Mutations in this gene disrupt mitochondrial membrane potential, leading to mitochondrial swelling and cell death^8,9^. Previous reports have indicated concurrent mutations in Arg217, either c.649C>T, p.(Arg217Cys) or c.650G>A(Arg217His)^8,9^. The amino acid change p.Arg217Cys seems to entail a gain of pathological function that interferes with the physiological *SLC25A24* function, regulating the mitochondrial permeability transition pore^8,9^.

Murayama et al. reported that *SLC25A24* is involved in secondary oxidative phosphorylation^3^. In the current case, the respiratory chain enzyme assay showed that the activities of complexes I and IV in muscle were decreased, especially that of complex I was less than 20%.

Bernier et al. showed the diagnostic criteria for MRCD^10^. MRCD can be diagnosed when two or more major criteria or one major plus two minor criteria are present. The major criteria they presented included enzyme activity in one organ being less than 20% of the standard and the identification of obvious pathogenic genes in nuclear or mitochondrial DNA. This patient exhibited a pathogenic variant in *SLC25A24* and a notable decrease in complex I activity in muscle. Meeting the two major criteria led to the diagnosis of neonatal MRCD based on these findings.

So far, 12 cases of FPS have been reported in the literature, but only two cases have been reported in ELBW infants. Among these cases, one involved an infant born at 35 weeks and weighing 800 g who died at 7 h of age, and the other involved an infant born at 32 weeks and weighing 866 g who died at 20 h of age. In both cases, the cause of death was respiratory distress^11,12^. In contrast, in the presented case, the infant was born at 767 g, and respiratory and circulatory failure were managed with intensive care. However, the infant died of sepsis at 15 days of age. Despite occasional reports of FPS at the age of 14 or 15 years, the prognosis is poor in cases of severe growth restriction from the fetal stage and in ELBW infants. In reviewing past reports, differences in severity and clinical features by mutation type were not apparent.

There have been no previous reports on hyperlactatemia in FPS. This patient was the most premature and had a low birth weight; therefore, we speculate that it was the most severe phenotype among all previous FPS reports. This case supports the idea that pathological gain of function variants in *SLC25A24* can cause disease, because these occur in hot spots regardless of population. In conclusion, we report a case of FPS with neonatal mitochondrial disease. To the best of our knowledge, this is the first reported case of FPS in Japan.

## References

[1] Ryu, J., Ko, J. M. & Shin, C. H. A 9-year-old Korean girl with Fontaine progeroid syndrome: a case report with further phenotypical delineation and description of clinical course during long-term follow-up. *BMC Med. Genet.* **20**, 188 (2019). 31775791 10.1186/s12881-019-0921-9 PMC6882017

[2] Lally, S. et al. Fontaine progeroid syndrome—a case report. *Clin. Case Rep.* **10**, e06291 (2022). 10.1002/ccr3.6291 PMC9448962 36093452

[3] Murayama, K., Shimura, M., Liu, Z., Okazaki, Y. & Ohtake, A. Recent topics: the diagnosis, molecular genesis, and treatment of mitochondrial diseases. *J. Hum. Genet.* **64**, 113–125 (2019). 30459337 10.1038/s10038-018-0528-6

[4] Stenton, S. L. & Prokisch, H. Advancing genomic approaches to the molecular diagnosis of mitochondrial disease. *Essays Biochem.*10.1042/EBC20170110 (2018). 29950319 10.1042/EBC20170110

[5] Rahman, J. & Rahman, S. Mitochondrial medicine in the omics era. *Lancet*10.1016/S0140-6736(18)30727-X (2018). 29903433 10.1016/S0140-6736(18)30727-X

[6] Richards, S. et al. Standards and guidelines for the interpretation of sequence variants: a joint consensus recommendation of the American College of Medical Genetics and Genomics and the Association for Molecular Pathlogy. *Genet. Med.* **17**, 405–424 (2015). 25741868 10.1038/gim.2015.30 PMC4544753

[7] Del Arco, A. & Satrustegui, J. Identification of a novel human subfamily of mitochondrial carriers with calcium-binding domains. *J. Biol. Chem.* **279**, 24701–24713 (2004). 15054102 10.1074/jbc.M401417200

[8] Writzl, K. et al. De novo mutations in SLC25A24 cause a disorder characterized by early aging, bone dysplasia, characteristic face, and early demise. *Am. J. Hum. Genet.* **101**, 844–855 (2017). 29100094 10.1016/j.ajhg.2017.09.017 PMC5673633

[9] Ehmke, N. et al. De novo mutations in SLC25A24 cause a craniosynostosis syndrome with hypertrichosis, progeroid appearance, and mitochondrial dysfunction. *Am. J. Hum. Genet.* **101**, 833–843 (2017). 29100093 10.1016/j.ajhg.2017.09.016 PMC5673623

[10] Bernier, F. P. et al. Diagnostic criteria for respiratory chain disorders in adults and children. *Neurology* **59**, 1406–1411 (2002). 12427892 10.1212/01.wnl.0000033795.17156.00

[11] Rodriguez, J. I., Perez-Alonso, P., Funes, R. & Perez-Rodriguez, J. Lethal neonatal Hutchinson–Gilford progeria syndrome. *Am. J. Med. Genet.* **82**, 242–248 (1999). 10215548

[12] Castori, M. et al. Fontaine–Farriaux syndrome: a recognizable craniosynostosis syndrome with nail, skeletal, abdominal, and central nervous system anomalies. *Am. J. Med. Genet. A* **149**, 2193–2199 (2009). 10.1002/ajmg.a.32763 19731360

